# Comparison of suprapatellar and infrapatellar intramedullary nailing for tibial shaft fractures: a systematic review and meta-analysis

**DOI:** 10.1186/s13018-018-0846-6

**Published:** 2018-06-14

**Authors:** Liqing Yang, Yuefeng Sun, Ge Li

**Affiliations:** Department of orthopedics, Shengjing Hospital of China Medical University, Shenyang, 110004 China

**Keywords:** Tibial shaft fractures, Infrapatellar, Suprapatellar, Intramedullary nail, Meta-analysis

## Abstract

**Background:**

Optimal surgical approach for tibial shaft fractures remains controversial. We perform a meta-analysis from randomized controlled trials (RCTs) to compare the clinical efficacy and prognosis between infrapatellar and suprapatellar intramedullary nail in the treatment of tibial shaft fractures.

**Methods:**

PubMed, OVID, Embase, ScienceDirect, and Web of Science were searched up to December 2017 for comparative RCTs involving infrapatellar and suprapatellar intramedullary nail in the treatment of tibial shaft fractures. Primary outcomes were blood loss, visual analog scale (VAS) score, range of motion, Lysholm knee scores, and fluoroscopy times. Secondary outcomes were length of hospital stay and postoperative complications. We assessed statistical heterogeneity for each outcome with the use of a standard *χ*^2^ test and the *I*^2^ statistic. The meta-analysis was undertaken using Stata 14.0.

**Results:**

Four RCTs involving 293 participants were included in our study. The present meta-analysis indicated that there were significant differences between infrapatellar and suprapatellar intramedullary nail regarding the total blood loss, VAS scores, Lysholm knee scores, and fluoroscopy times.

**Conclusion:**

Suprapatellar intramedullary nailing could significantly reduce total blood loss, postoperative knee pain, and fluoroscopy times compared to infrapatellar approach. Additionally, it was associated with an improved Lysholm knee scores. High-quality RCTs were still required for further investigation.

## Background

Tibial shaft fracture is common and comprises about 2% of workload in all fractures in adult [1, 2]. The intramedullary nail (IMN) fixation is reported to be a successful surgical procedure for the treatment of tibial shaft fracture and shows improved outcome in functional recovery [3]. The traditional infrapatellar is the common surgical approach to insert an IMN for the tibial shaft fracture. However, this approach requires a flexed knee and makes it difficult to use correctly in proximal third tibial shaft fractures, because the quadriceps muscle forces the proximal fragment into extension, resulting in deformities of angulation and fragment displacement [4]. Additionally, chronic postoperative knee pain is one of the most frequent complications after IMN insertion; the incidence was reported varying from 10 to 80% [5, 6].

The semiextended approach for tibial IMN insertion was first introduced in 2000 and then was modified into suprapatellar approach [7]. It has more simple access to entry point at proximal tibia, facilitates fracture reduction, and avoids patellar tendon. Additionally, the extended position of the lower limb allows for easier fluoroscopic imaging [4]. Zhan et al. reported that suprapatellar approach may be effective in reducing the incidence of postoperative knee pain and prevent degenerative disorder of knee joint [8]. Besides, it can also decrease the risk of intraoperative second shifting. However, some studies showed that intraarticular injury may be the potential complication of this technique.

Currently, the comparison of infrapatellar and suprapatellar approach for tibial IMN insertion is rarely reported, and most of them are retrospective studies. The optional surgical approach remains controversial. Therefore, we perform a systematic review and meta-analysis of randomized controlled trials (RCTs) to compare the clinical efficacy and prognosis between classical infrapatellar and suprapatellar IMN in the treatment of tibial shaft fractures. We hypothesize that suprapatellar approach is superior to infrapatellar approach in terms of functional outcome, postoperative pain, and complications.

## Methods

Ethical approval for this study was deemed unnecessary because it was a review of existing literature and did not involve any handling of individual patient’s data.

### Search methodology

Two reviewers independently searched PubMed, OVID, Embase, ScienceDirect, and Web of Science. All databases were searched up to December 2017, without restrictions on publication date and language. The terms were used to search the databases: “tibia shaft fracture,” “intramedullary nail,” “infrapatellar,” and “suprapatellar.” Search terms were combined using the Boolean operators “AND” or “OR.” Reference lists of relevant articles were manually searched to identify additional trials.

### Inclusion and exclusion criteria

Studies were considered eligible when they met following criteria: (1) published clinical RCTs; (2) patients with tibial shaft fracture, intervention groups received infrapatellar approach IMN, and control groups received suprapatellar approach IMN; and (3) studies with at least one of the following outcomes: blood loss, visual analog score (VAS), range of motion, Lysholm knee scores, fluoroscopy times, length of hospital stay, and postoperative complications. Studies would be excluded from present meta-analysis for incomplete data, case reports, conference abstract, or review articles.

### Study selection

Two investigators independently selected articles according to the criteria described above. The full text was scanned to determine whether articles fit the inclusion criteria. We resolved disagreements by discussion until a consensus was search. If no consensus was reached, a third investigator was consulted.

### Data extraction

Two investigators independently extracted the data from the eligible studies that met the inclusion criteria. A double-check procedure was performed to test the accuracy of the extracted data. The information extracted from the studies were as follows: first author names, publishing year, study design, sample size, age, gender, intervention of each groups, duration of follow-up, and outcome measures. Primary outcomes were blood loss, visual analog scale (VAS) score, range of motion, Lysholm knee scores, and fluoroscopy times. Secondary outcomes were length of hospital stay and postoperative complications. Corresponding authors were consulted to obtain incomplete outcome data.

### Data analysis

We performed all meta-analysis using the Stata 14.0 software. For continuous outcomes, the number of patients, means, and standard deviations were pooled to a weighted mean difference (WMD) and a 95% confidence interval (CI). For dichotomous outcomes, the risk difference (RD) and the 95% CI were assessed. The assessment for statistical heterogeneity was calculated using the chi-square and *I*-square tests. A fixed-effects model was used when *I*^2^ < 50% and *P* > 0.05; otherwise, the random-effects model was adopted.

### Quality assessment

A quality assessment of each RCT was performed according to the Cochrane Handbook for Systematic Reviews of Interventions. Two authors independently evaluated the risk of bias of the included RCTs based on the following items: random sequence generation, allocation concealment, blinding, incomplete outcome data, selective reporting, and other sources of bias. The evidence grade was assessed using the guidelines of the GRADE (Grading of Recommendations, Assessment, Development, and Evaluation) working group including the following items: risk of bias, inconsistency, indirectness, imprecision, and publication bias. The recommendation level of evidence was classified into the following categories: (1) high, which means that further research is unlikely to change confidence in the effect estimate; (2) moderate, which means that further research is likely to significantly change confidence in the effect estimate but may change the estimate; (3) low, which means that further research is likely to significantly change confidence in the effect estimate and to change the estimate; and (4) very low, which means that any effect estimate is uncertain. GRADE pro version 3.6 software is used for the evidence synthesis.

## Results

### Search result

**Fig. 1:**
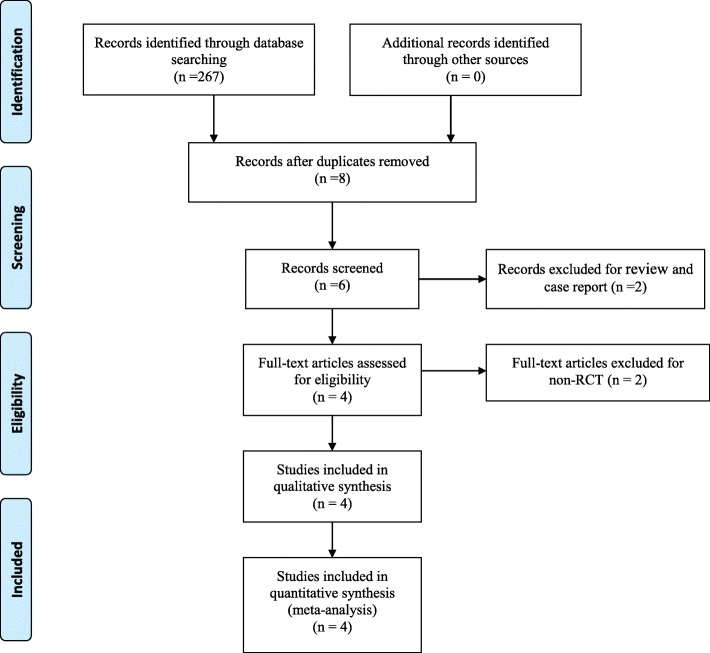
Search results and the selection procedure

A total of 267 studies related to IMN and tibial shaft fractures were reviewed. After reading the titles and abstracts, 263 studies were excluded from the present meta-analysis. Four RCTs [9–12] which published between 2015 and 2017 eventually satisfied the eligibility criteria for this study. There were 142 participants in the experimental groups and 151 patients in the control groups. The search process was proceeded as presented in Fig. 1.

### Study characteristics

All of the included studies were RCTs. There were two RCTs performed in China and one each in the USA and India. All RCTs had defined eligibility criteria. The sample size ranged from 25 to 162 and average age ranged from 40 to 47 years old. In these studies, the experimental groups received infrapatellar approach IMN for tibial shaft fractures and the control groups received suprapatellar approach IMN. Duration of follow-up ranged from 6 to 16 months. The characteristics of the included studies are shown in Table 1.

**Table 1:** Trial characteristics

Author	Study design	Location	Sample size	Mean age	Female patient	Type of fractures	IP group	SP group	Follow-up
			(IP/SP)	(IP/SP)	(IP/SP)				
Chan, 2015	RCT	USA	14/11	43/40	4/5	Open tibial shaft fractures: 3 Closed tibial shaft fractures: 22	Infrapatellar tibial nail insertion	Suprapatellar tibial nail insertion	16 months
Zhe, 2016	RCT	China	30/38	46/42	4/3	Open tibial shaft fractures: 8 Closed tibial shaft fractures: 60	Infrapatellar intramedullary nailing	Suprapatellar intramedullary nailing	6 months
Sun, 2016	RCT	China	81/81	47/46	16/15	Open tibial shaft fractures: 21 Closed tibial shaft fractures: 141	Infrapatellar intramedullary nailing	Suprapatellar intramedullary nailing	24 months
Sreekumar, 2017	RCT	India	17/21	44/42	9/8	Open tibial shaft fractures: 5 Closed tibial shaft fractures: 33	Infrapatellar tibial nail	Suprapatellar tibial nail	12 months

*IP* infrapatellar, *SP* suprapatellar, *RCT* randomized controlled trial

### Risk of bias

**Fig. 2:**
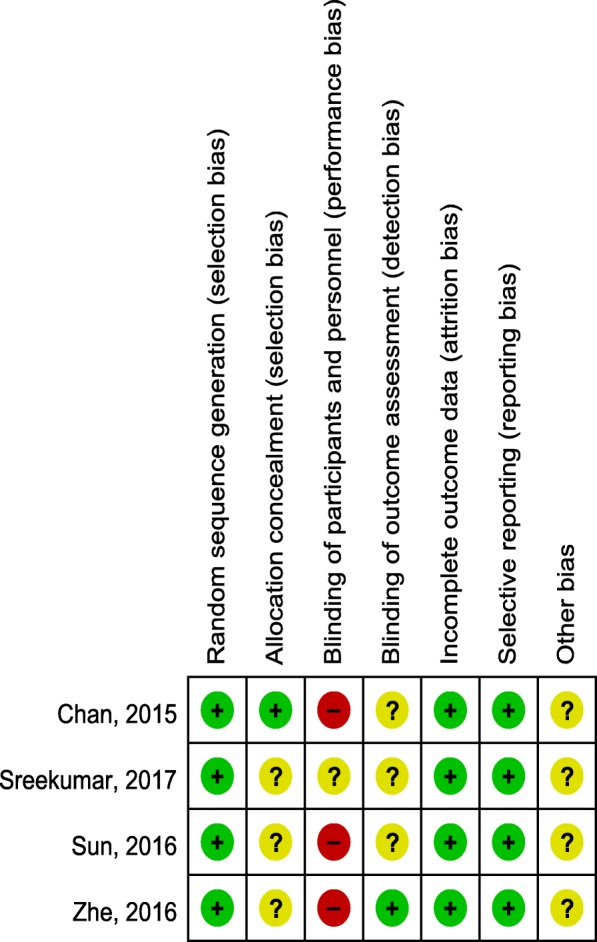
Methodological quality of the randomized controlled trials

**Fig. 3:**
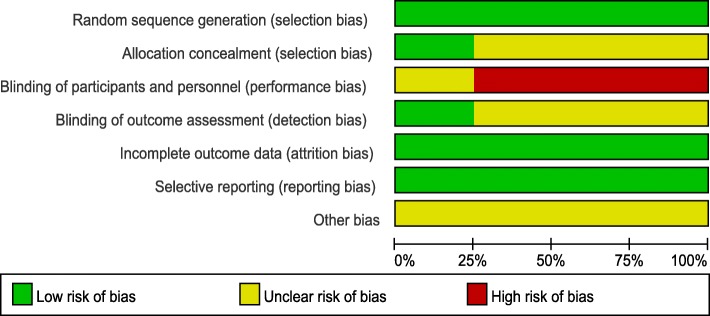
Risk of bias

Seven aspects of the RCTs related to the risk of bias were assessed, following the instructions in the Cochrane Handbook for Systematic Reviews of Interventions (Fig. 2). Randomization was performed in all RCTs and all of them mentioned that the list of random numbers were generated from a computer. Only one [9] article used sealed envelopes for allocation concealment. None RCTs reported double blinding to the surgeons and participants. One study [10] showed that assessor was blinded. Low risk of bias due to incomplete outcome data and selective outcome reporting were detected. Judgments regarding each risk of bias item were presented as percentages across all the included RCTs in Fig. 3.

### Outcome analysis

#### Total blood loss

**Fig. 4:**
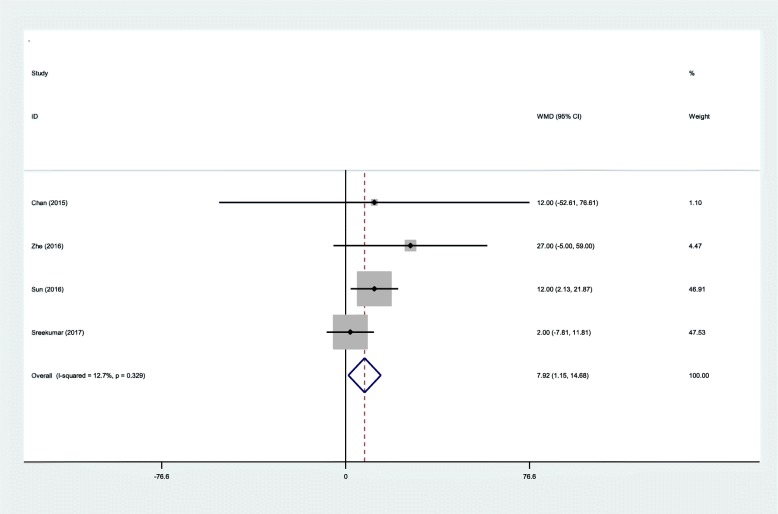
Forest plot diagram showing effect of IP versus SP on total blood loss

All RCTs reported the total blood loss following IMN fixation. No statistical heterogeneity was observed in our study (*χ*^2^ = 3.44, df = 3, *I*^2^ = 12.7%, *P* = 0.329); therefore, a fixed-effects model was applied. There was significant difference between the infrapatellar groups and suprapatellar groups regarding the total blood loss (WMD = 7.92, 95% CI 1.15 to 14.68, *P* = 0.022; Fig. 4).

#### VAS scores

**Fig. 5:**
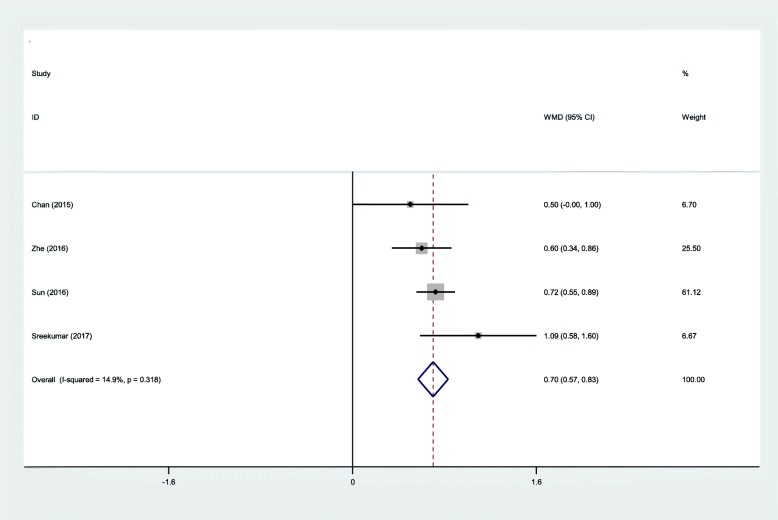
Forest plot diagram showing effect of IP versus SP on VAS scores

All RCTs showed the postoperative VAS scores at the first follow-up after IMN fixation. There was no significant heterogeneity (*χ*^2^ = 3.52, df = 3, *I*^2^ = 14.9%, *P* = 0.318); therefore, a fixed-effects model was adopted. The result of meta-analysis indicated that suprapatellar groups was associated with a significant reduction in the VAS scores (WMD = 0.70, 95% CI 0.570 to 0.83, *P* = 0.000; Fig. 5).

#### Range of motion

**Fig. 6:**
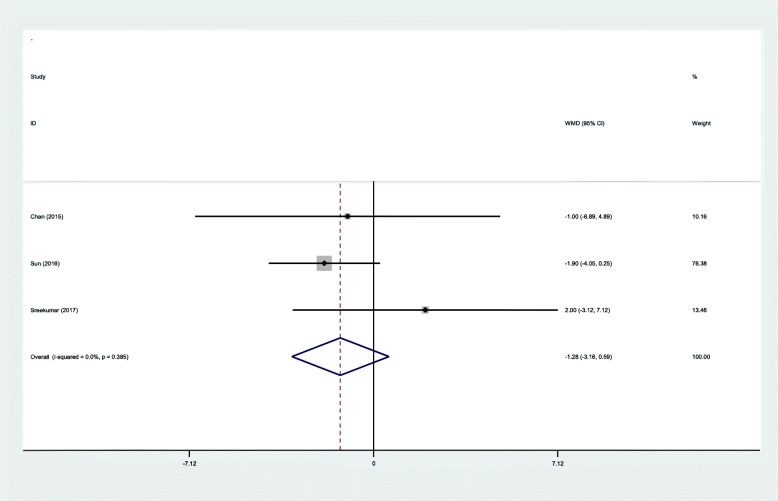
Forest plot diagram showing effect of IP versus SP on range of motion

Three RCTs provided the outcome of range of motion after IMN fixation. There was no significant heterogeneity (*χ*^2^ = 1.91, df = 2, *I*^2^ = 0%, *P* = 0.385) and a fixed-effects model was used. The pooled results demonstrated that there was no significant difference between two groups regarding the range of motion (WMD = − 1.28, 95% CI − 3.16 to 0.59, *P* = 0.180; Fig. 6).

#### Lysholm knee scores

**Fig. 7:**
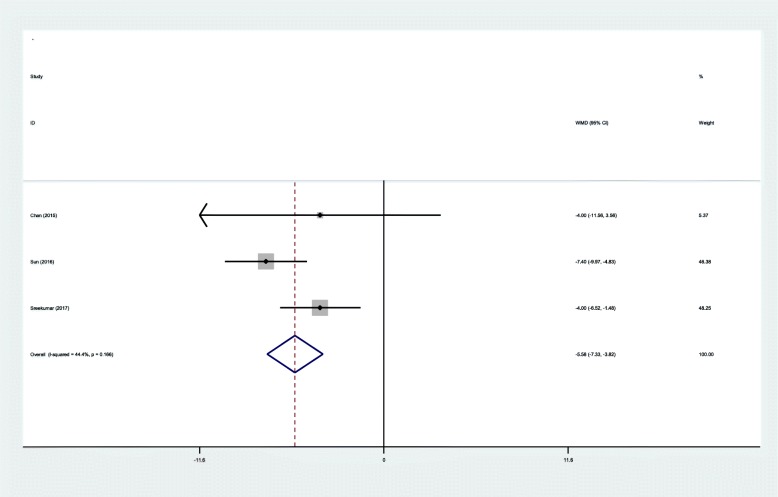
Forest plot diagram showing effect of IP versus SP on Lysholm knee scores

Lysholm knee scores were reported in three RCTs. There was no significant heterogeneity (*χ*^2^ = 3.60, df = 2, *I*^2^ = 44.4%, *P* = 0.166), and a fixed-effects model was used. The present meta-analysis revealed that there was significant difference between two groups in terms of Lysholm knee scores (WMD = − 5.58, 95% CI − 7.33 to − 3.83, *P* = 0.000; Fig. 7).

#### Fluoroscopy times

**Fig. 8:**
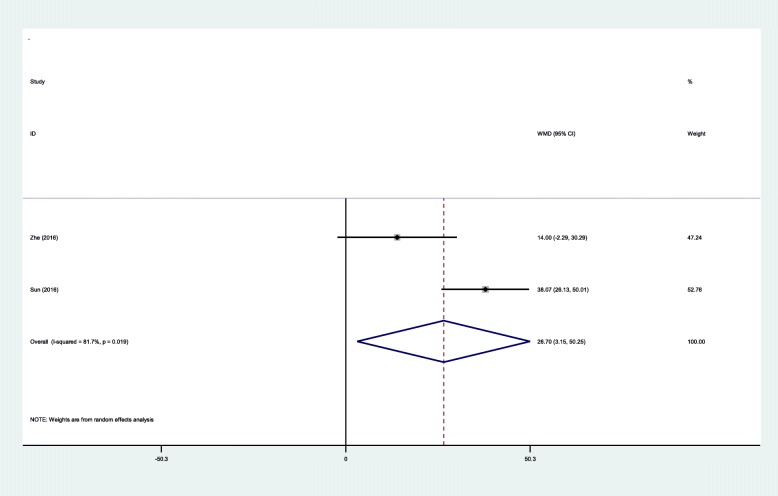
Forest plot diagram showing effect of IP versus SP on fluoroscopy times

Two RCTs showed the fluoroscopy times during surgery. Significant statistical heterogeneity was found (*χ*^2^ = 5.46, df = 1, *I*^2^ = 81.7%, *P* = 0.019), and a random-effects model was applied. There was significant difference between groups regarding the total fluoroscopy times (WMD = 26.70, 95% CI 3.15 to 50.25, *P* = 0.026; Fig. 8).

#### Length of hospital stay

**Fig. 9:**
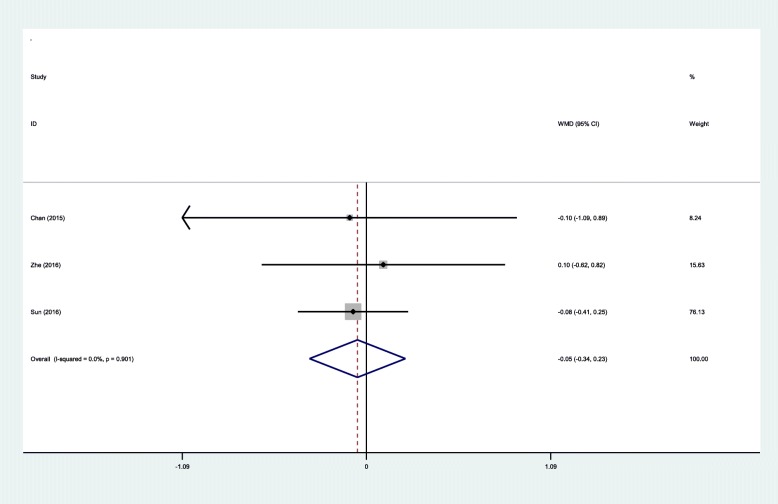
Forest plot diagram showing effect of IP versus SP on length of hospital stay

Three RCTs showed the length of hospital stay. A fixed-effects model was adopted because no significant heterogeneity was found (*χ*^2^ = 0.21, df = 2, *I*^2^ = 0%, *P* = 0.901). There was no significant difference between the two groups in terms of length of hospital stay (WMD = − 0.05, 95% CI − 0.33 to 0.23, *P* = 0.713; Fig. 9).

#### Postoperative complications

**Fig. 10:**
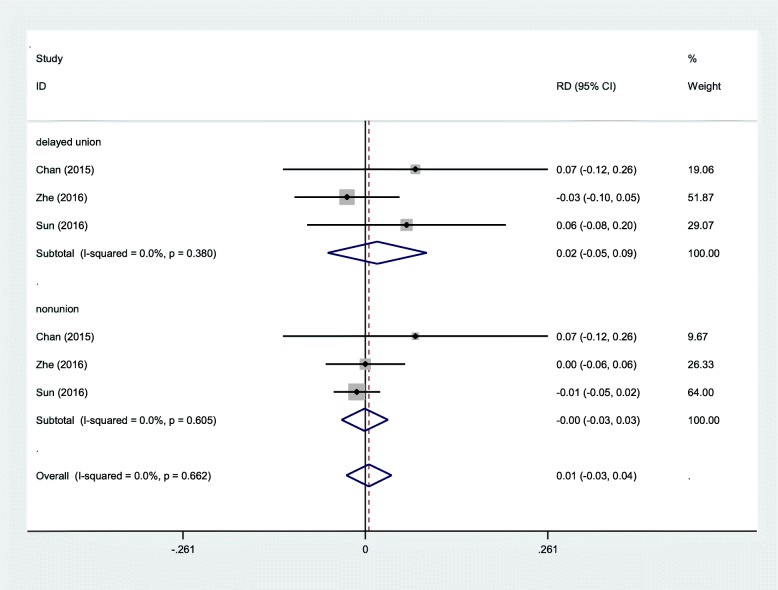
Forest plot diagram showing effect of IP versus SP on postoperative complications

Three RCTs reported the postoperative complications including nonunion or delayed union. A fixed-effects model was adopted (*χ*^2^ = 3.25, df = 5, *I*^2^ = 0%, *P* = 0.662). There was no significant difference between groups regarding the incidence of the postoperative complications (RD = 0.01, 95% CI − 0.03 to 0.04, *P* = 0.755; Fig. 10).

#### Evidence level and recommendation strengths

Quality of evidence was evaluated by the GRADE system. The evidence quality for each outcome was moderate to high. Therefore, we agreed that the overall evidence quality was moderate, which means that further research is likely to significantly change confidence in the effect estimate but may change the estimate (Table 2).

**Table 2:** The GRADE evidence quality

Quality assessment						No. of patients		Effect	Quality	Importance
No. of studies	Design	Limitations	Inconsistency	Indirectness	Imprecision	IP groups	SP groups			
Total blood loss										
4	RCT	Serious limitations	No serious inconsistency	No serious indirectness	No serious imprecision	142	151	WMD = 7.92, 95% CI 1.15 to 14.68	High	Critical
VAS scores										
4	RCT	Serious limitations	No serious inconsistency	No serious indirectness	No serious imprecision	142	151	WMD = 0.70, 95% CI 0.570 to 0.83	High	Critical
Range of motion										
3	RCT	Serious limitations	No serious inconsistency	No serious indirectness	No serious imprecision	112	113	WMD = − 1.28, 95% CI − 3.16 to 0.59	High	Critical
Lysholm knee scores										
3	RCT	Serious limitations	No serious inconsistency	No serious indirectness	No serious imprecision	112	113	WMD = − 5.58, 95% CI − 7.33 to − 3.83	High	Critical
Fluoroscopy times										
2	RCT	Serious limitations	Serious inconsistency	No serious indirectness	No serious imprecision	111	119	WMD = 26.70, 95% CI 3.15 to 50.25	Moderate	Critical
Length of hospital stay										
3	RCT	Serious limitations	No serious inconsistency	No serious indirectness	No serious imprecision	125	130	WMD = − 0.05, 95% CI − 0.33 to 0.23	High	Critical
Postoperative complications										
3	RCT	Serious limitations	No serious inconsistency	No serious indirectness	No serious imprecision	125	130	RD = 0.01, 95% CI − 0.03 to 0.04	High	Critical

#### Publication bias

**Fig. 11:**
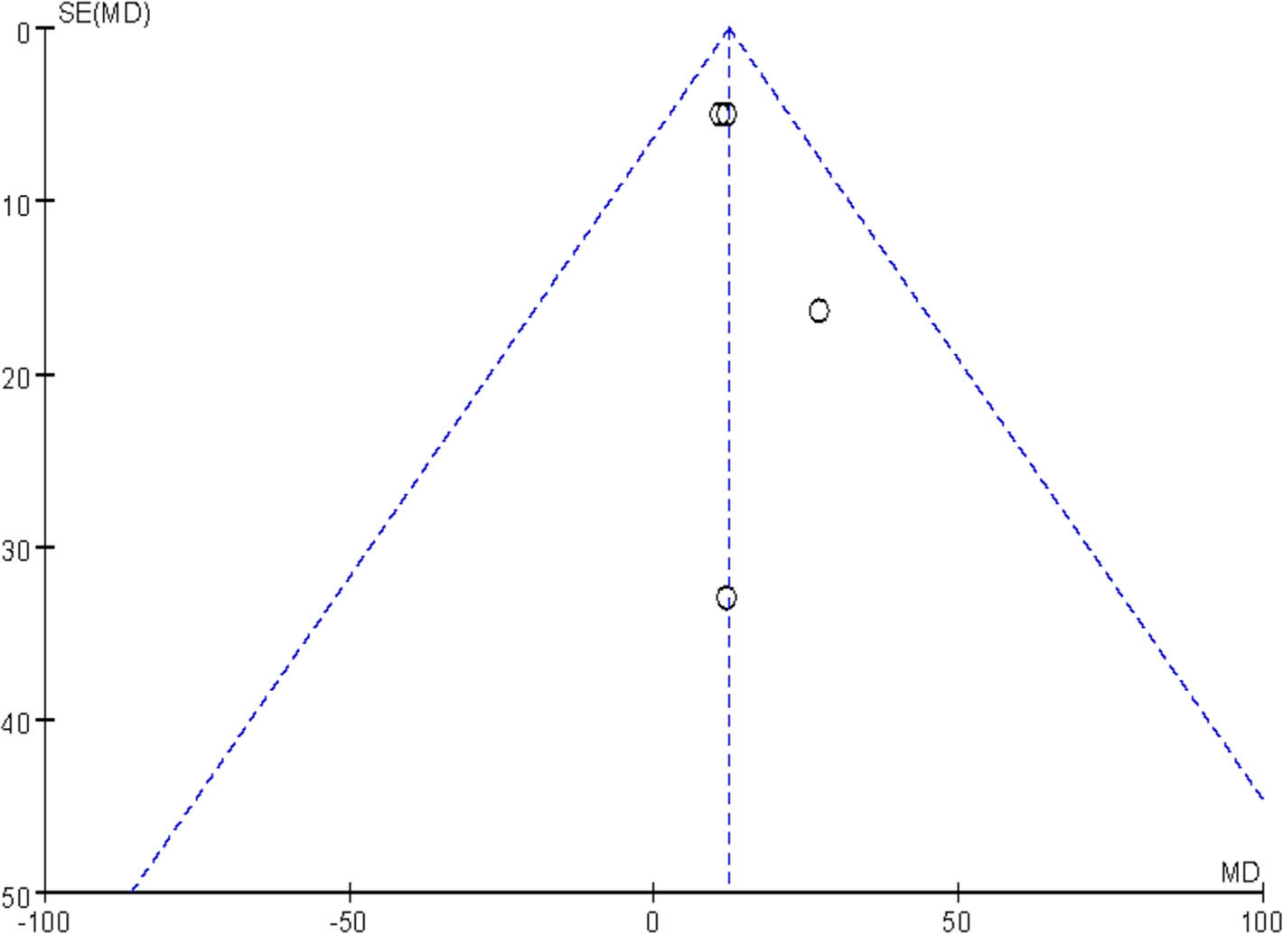
A funnel plot of total blood loss

**Fig. 12:**
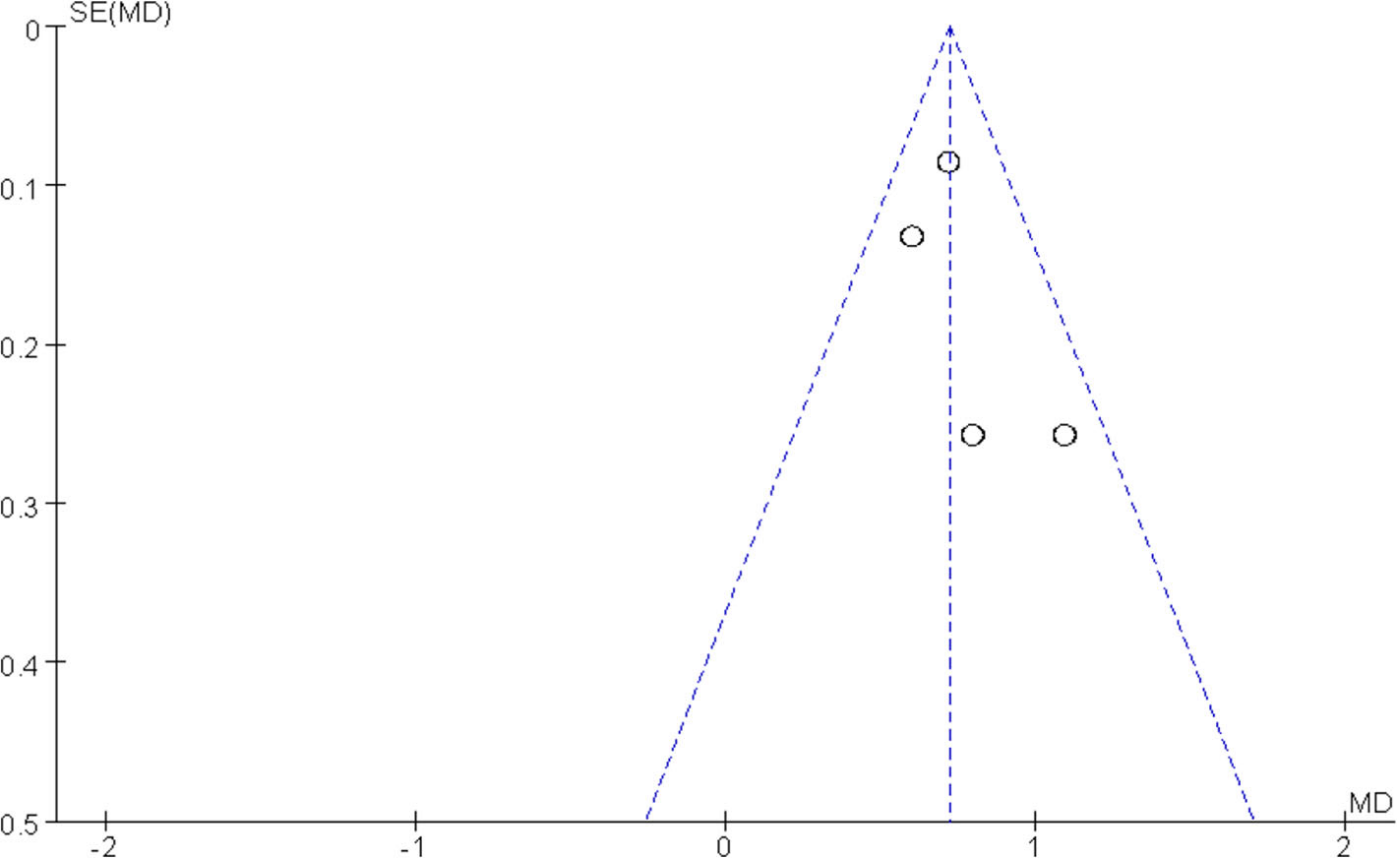
A funnel plot of VAS scores

Publication bias was assessed by a funnel plot diagram. The funnel plot diagrams of total blood loss and VAS scores were symmetrical, indicating a low risk of publication bias (Figs. 11 and 12).

## Discussion

To the best of our knowledge, it was the first meta-analysis from RCTs to compare the clinical and functional outcomes of the knee joint after infrapatellar versus suprapatellar tibial nail insertion. The most important finding of the present meta-analysis was that suprapatellar approach of IMN was associated with a significant reduction in total blood, VAS scores, and fluoroscopy times compared with infrapatellar approach. Additionally, there were significant differences between groups regarding the Lysholm knee scores. However, there was no evidence that suprapatellar approach was associated with a lower incidence of joint degeneration of the patellofemoral joint. Further research was still required. The overall evidence quality was moderate, which means that further research is likely to significantly change confidence in the effect estimate but may change the estimate.

Tibial shaft fractures were common in long bone and were usually caused by high-energy trauma such as traffic accidents and falling from a height [13, 14]. The IMN was considered the gold standard for the treatment of tibial shaft fractures with advantages of preferred stable fixation and less damage to vascularity and soft tissue [15]. Suprapatellar did not injure the tendon and was considered popular surgical approach [16]. Additionally, suprapatellar IMN could insert nail with knee extended and avoid the risk of infrapatellar nerve damage. Reducing perioperative blood loss was an important issue which may promote recovery and decrease the transfusion requirements. Few RCTs reported the total blood loss between various surgical approaches in tibial shaft fractures. The present meta-analysis revealed that suprapatellar approach was associated with a significant reduction of total blood loss.

Effective pain management may improve patients’ satisfaction and decrease postoperative complications. Postoperative pain following intramedullary nailing surgery was the major concern, and patients often complained of moderate to severe pain [17, 18]. It may be caused by the injury of the knee structure and nerve. Besides, surgical stress response which included inflammatory components also induced postoperative pain. Leliveld and Verhofstad [19] reported that 38% patients who underwent infrapatellar incision had complication of chronic knee pain and the incidence of iatrogenic damage to the infrapatellar nerve after IMN was high and lasting. Injury to this nerve appeared to be associated with postoperative knee pain. The suprapatellar approach was performed by an incision which was proximal to the patella, and the intramedullary nail passed through trochlear groove. Theoretically, there was no risk of injury to the patellar tendon and the infrapatellar nerve. Courtney et al. [20] reported that the infrapatellar nerve could be well protected with suprapatellar approach. Gaines et al. [21] showed that there was a higher risk of articular structure damage with infrapatellar approach than with suprapatellar approach; however, there was no significant statistical difference. Based on the current controversy, we performed this meta-analysis from published RCTs and indicated that there was a lower incidence of knee pain with suprapatellar approach compared to infrapatellar insertion.

Decreased range of motion after IMN was an undesirable outcome and was well documented in studies and was varied [22]. Multiple factors may affect the range of motion such as the damage to the vascularity and soft tissue. However, different surgical approach for tibial shaft fractures remains controversial. Leliveld and Verhofstad [19] reported that knee range of motion was equivalent to the unaffected side with infrapatellar tibial IMN on long-term follow-up. Though Chan et al. [9] showed an improved range of motion with suprapatellar approach compared with infrapatellar approach, there was no significant difference. Our study observed no significant statistical difference. Long-term follow-up was required.

Lysholm et al. [23] published their first knee scoring scale in 1982. It was a questionnaire that contained eight items about the function and symptom of knee, which described a validated evaluation of patient activities of daily living. It has been widely used in various types of knee fractures. Song et al. [24] showed that there was a closely relationship between Lysholm knee scores and knee pain in patients undergoing tibial IMN. Our study indicated that there was an improved Lysholm knee scores in suprapatellar groups compared with infrapatellar groups. Fluoroscopy time was significantly shorter in suprapatellar groups. The infrapatellar position made it difficult to perform a fluoroscopy during the surgical procedure. Capturing the orthogonal view of tibia was much easier with knee in semi-extended position, and this position may simplify the reduction of the fracture [25].

Several potential limitations of the present meta-analysis should be noted: (1) only four RCTs were included in our study, and the sample sizes were small; thus, it may result in overestimating the outcomes; (2) methodological weakness existed in all RCTs which may influence the results; (3) due to the limited studies, we failed to perform a subgroup analyses to investigate the other factors, such as gender, age, body mass index, and fracture type; thus, we could not determine the source of heterogeneity; (4) short-term follow-ups may lead to an underestimation of complications; and (5) all included RCTs were English and Chinese publications; thus, publication bias was unavoidable.

## Conclusion

Suprapatellar intramedullary nailing could significantly reduce total blood loss, postoperative knee pain, and fluoroscopy times compared to infrapatellar approach. Additionally, it was associated with an improved Lysholm knee scores. High-quality RCTs were still required for further investigation.

## Abbreviations

IMN: Intramedullary nail
RCT: Randomized controlled trials
VAS: Visual analog score

## References

[1] Larsen P, Lund H, Laessoe U, Graven-Nielsen T, Rasmussen S (2014). Restrictions in quality of life after intramedullary nailing of tibial shaft fracture: a retrospective follow-up study of 223 cases. J Orthop Trauma.

[2] Court-Brown CM, Caesar B (2006). Epidemiology of adult fractures: a review. Injury.

[3] Ahmad N, Khan MS, Afridi SA, Afridi SA, Awan AS, Afridi SK, Sultan S, Saifullah K, Lodhi FS (2016). Efficacy and safety of interlocked intramedullary nailing for open fracture shaft of tibia. Journal of Ayub Medical College, Abbottabad : JAMC.

[4] Zelle BA, Boni G (2015). Safe surgical technique: intramedullary nail fixation of tibial shaft fractures. Patient safety in surgery.

[5] Lefaivre KA, Guy P, Chan H, Blachut PA (2008). Long-term follow-up of tibial shaft fractures treated with intramedullary nailing. J Orthop Trauma.

[6] Toivanen JA, Vaisto O, Kannus P, Latvala K, Honkonen SE, Jarvinen MJ (2002). Anterior knee pain after intramedullary nailing of fractures of the tibial shaft. A prospective, randomized study comparing two different nail-insertion techniques. J Bone Joint Surg Am.

[7] Hernigou P, Cohen D (2000). Proximal entry for intramedullary nailing of the tibia. The risk of unrecognised articular damage. The Journal of bone and joint surgery British volume.

[8] Zhendong H, Li J, Hu Z, Orthopedics T. Comparison of therapeutic effects of suprapatellar approach and infrapatellar approach intramedullary nail for tibial shaft fractures. J Pract Orthop. 2017;23(9):794–7

[9] Chan DS, Serrano-Riera R, Griffing R, Steverson B, Infante A, Watson D, Sagi HC, Sanders RW (2016). Suprapatellar versus infrapatellar tibial nail insertion: a prospective randomized control pilot study. J Orthop Trauma.

[10] Wang Z, Li S, Wang X, Tang X (2016). Supra-patellar versus infra-patellar intramedullary nailing in treatment of tibial shaft fractures. Chin J Orthop Trauma.

[11] Sun Q, Nie X, Gong J, Wu J, Li R, Ge W, Cai M (2016). The outcome comparison of the suprapatellar approach and infrapatellar approach for tibia intramedullary nailing. Int Orthop.

[12] Sreekumar K, Sreekumar K (2017). Suprapatellar versus infrapatellar tibial nail insertion: a prospective randomized control pilot study. J Evid Based Healthc.

[13] Guruprasad Y, Chauhan DS (2012). Tibial shaft fracture following graft harvestment for nasal augmentation. National journal of maxillofacial surgery.

[14] Anandasivam NS, Russo GS, Swallow MS, Basques BA, Samuel AM, Ondeck NT, Chung SH, Fischer JM, Bohl DD, Grauer JN (2017). Tibial shaft fracture: a large-scale study defining the injured population and associated injuries. Journal of clinical orthopaedics and trauma.

[15] Bode G, Strohm PC, Sudkamp NP, Hammer TO (2012). Tibial shaft fractures—management and treatment options. A review of the current literature. Acta Chir Orthop Traumatol Cechoslov.

[16] Morandi M, Banka T, Gaiarsa GP, Guthrie ST, Khalil J, Hoegler J, Lindeque BG (2010). Intramedullary nailing of tibial fractures: review of surgical techniques and description of a percutaneous lateral suprapatellar approach. Orthopedics.

[17] Song SY, Chang HG, Byun JC, Kim TY (2012). Anterior knee pain after tibial intramedullary nailing using a medial paratendinous approach. J Orthop Trauma.

[18] Jang Y, Kempton LB, TO MK, Sorkin AT (2017). Insertion-related pain with intramedullary nailing. Injury.

[19] Leliveld MS, Verhofstad MH (2012). Injury to the infrapatellar branch of the saphenous nerve, a possible cause for anterior knee pain after tibial nailing?. Injury.

[20] Courtney PM, Boniello A, Donegan D, Ahn J, Mehta S (2015). Functional knee outcomes in infrapatellar and suprapatellar tibial nailing: does approach matter?. Am J Orthop.

[21] Gaines RJ, Rockwood J, Garland J, Ellingson C, Demaio M (2013). Comparison of insertional trauma between suprapatellar and infrapatellar portals for tibial nailing. Orthopedics.

[22] Keating JF, O'Brien PI, Blachut PA, Meek RN, Broekhuyse HM (1997). Reamed interlocking intramedullary nailing of open fractures of the tibia. Clin Orthop Relat Res.

[23] Lysholm J, Gillquist J (1982). Evaluation of knee ligament surgery results with special emphasis on use of a scoring scale. Am J Sports Med.

[24] Song SY, Chang HG, Byun JC (2012). Anterior knee pain after tibial IMN using a medial paratendinous approach. J Orthop Trauma.

[25] Brink O (2016). Suprapatellar nailing of tibial fractures: surgical hints. Current orthopaedic practice.

